# Identification of the Substrate Recognition and Transport Pathway in a Eukaryotic Member of the Nucleobase-Ascorbate Transporter (NAT) Family

**DOI:** 10.1371/journal.pone.0041939

**Published:** 2012-07-25

**Authors:** Vasiliki Kosti, George Lambrinidis, Vassilios Myrianthopoulos, George Diallinas, Emmanuel Mikros

**Affiliations:** 1 Faculty of Biology, University of Athens, Panepistimiopolis, Athens, Greece; 2 School of Pharmacy, University of Athens, Panepistimiopolis, Athens, Greece; University of Cambridge, United Kingdom

## Abstract

Using the crystal structure of the uracil transporter UraA of *Escherichia coli*, we constructed a 3D model of the *Aspergillus nidulans* uric acid-xanthine/H^+^ symporter UapA, which is a prototype member of the Nucleobase-Ascorbate Transporter (NAT) family. The model consists of 14 transmembrane segments (TMSs) divided into a core and a gate domain, the later being distinctly different from that of UraA. By implementing Molecular Mechanics (MM) simulations and quantitative structure-activity relationship (SAR) approaches, we propose a model for the xanthine-UapA complex where the substrate binding site is formed by the polar side chains of residues E356 (TMS8) and Q408 (TMS10) and the backbones of A407 (TMS10) and F155 (TMS3). In addition, our model shows several polar interactions between TMS1-TMS10, TMS1-TMS3, TMS8-TMS10, which seem critical for UapA transport activity. Using extensive docking calculations we identify a cytoplasm-facing substrate trajectory (D360, A363, G411, T416, R417, V463 and A469) connecting the proposed substrate binding site with the cytoplasm, as well as, a possible outward-facing gate leading towards the substrate major binding site. Most importantly, re-evaluation of the plethora of available and analysis of a number of herein constructed UapA mutations strongly supports the UapA structural model. Furthermore, modeling and docking approaches with mammalian NAT homologues provided a molecular rationale on how specificity in this family of carriers might be determined, and further support the importance of selectivity gates acting independently from the major central substrate binding site.

## Introduction

The Nucleobase-Ascorbate Transporter (NATs) family, also called Nucleobase-Cation Symporter-2 (NCS2) family, is one of the most conserved carrier families, including hundreds of members in all organisms, prominent exceptions being *Saccharomyces cerevisiae* and protozoa [1], [2]. The function and specificity of nearly 20 NAT proteins, coming from bacteria, fungi, plants and mammals, is presently known, showing that most of them are specific for the symport of xanthine, uric acid or uracil with H^+^. In primates however, NAT homologues (SVCT1 and SVCT2) are specific for the co-transport of L-ascorbic acid/Na^+^ [1], [2]. Interestingly, none of the known NATs can recognise salvageable purines (adenine, guanine or hypoxanthine), cytosine or nucleosides. While in microorganisms NATs are not essential for life, serving mostly as nutrient scavengers for nucleobases, their function is necessary for normal growth and survival in plants and mammals [3], [4].

The UapA transporter of the filamentous ascomycetes *Aspergillus nidulans* is the prototype member of the NAT family, being one of the most extensively studied eukaryotic carriers with respect to regulation of expression and structure–function relationships. This is not only because of historical reasons, as *uapA* was among the first eukaryotic transporter genes identified genetically [5] and cloned [6], [7] but mainly due to the fact that *uapA* mutants can be easily selected or constructed through classical or reverse genetics, and subsequently analysed biochemically in great detail with simple kinetic studies. The wild-type transporter was shown to be highly specific for the uptake of xanthine and uric acid, as both substrates are recognised with high affinity (7–8 µΜ) and transported with high capacity [8]–[10]. In addition, several analogues of xanthine or uric acid, especially those that do not have modifications in positions N1-H, N7-H or N9 of the purine ring, were shown to act as substrates or ligands, albeit with lower affinity [10], [11]. Through the analysis of more than a hundred UapA mutants, especially those affecting the specificity or the transport kinetics, the functional importance of several residues has been established [1], [2], [12], [13]. Four absolutely conserved amino acid residues (Q85, E356, D388 and N409) are irreplaceable for function. Among these residues, E356 was proposed to form direct contacts with the purine ring, based on the fact that a specific mutation (E356D) dramatically increases the binding of physiological substrates but reduces their transport [11]. A second partially conserved amino acid, Q408, was also proposed to be involved in direct contacts with substrates, because its substitution with Pro dramatically reduces binding of the physiological substrates, but mostly because its substitution with Glu offers UapA the capability of recognizing novel substrates, such as hypoxanthine and guanine [9]. Furthermore, a kinetic analysis using several xanthine analogues as competitive inhibitors of xanthine uptake suggested that E356 and Q408 might interact with N1H and N9 or N7H of the purine ring [11]. Four additional absolutely or partially conserved residues, H86, G411, T417, R418, were also shown to be crucial for determining the transport activity of UapA [9], [10], [14]. Importantly, reverse genetics and Cys-scanning mutational analysis of the homologous XanQ xanthine transporter of *Escherichia coli* showed that the same residues as those found essential for UapA function are also critical for the activity of the bacterial carrier [15]–[18]. Most interestingly, randomly selected specificity mutations enlarging the substrate profile of UapA concerned nine partially or non-conserved residues, namely N71, Q113, F406, A441, V463, A469, R481, T526 and F528, distributed in several regions of UapA structure [11], [12], [19], [20]. None of these residues was however critical for the binding and transport efficiency of the physiological substrates of UapA.

The crystallization of the first NAT homologue from *Escherichia coli,* the uracil transporter UraA [21], allowed us to build a preliminary topological model of UapA and to verify the topology of the residues affecting UapA function and specificity [13]. The 3D model of UapA corresponds to a cytoplasm-facing conformer made of 14 transmembrane segments (TMSs) divided into two inverted repeats (TMS1–7 and TMS8–14). The structure is spatially arranged into a core and a gate domain, consisting of TMS1–4/8–11 and TMS5–7/12–14, respectively. All residues essential or critical for UapA function fall within TMS1, TMS3 and TMS10 in the core domain. More importantly, residues E356 (TMS8) and Q408 (TMS10) in UapA correspond to residues E241 and E290 in UraA, which were shown to interact with the uracil. The UapA model also revealed putative critical interactions of TMS1 with both TMS3 and TMS10. The importance of TMS3 and the interaction of TMS1 with TMS3 were genetically supported by characterising second-site suppressors of the H86D mutation (TMS1), which are located in M151 (TMS3). Thus, both in UraA and UapA the substrate binding site seems to be built by specific residues in TMS3, TMS8 and TMS10. A similar conclusion was drawn by a recently published 3D model of the XanQ permease [22]. Interestingly, the preliminary UapA model also confirmed that all specificity mutations which do not affect the kinetics of transport of physiological substrates are located distantly from the proposed binding site, that is, outside TMS1, TMS3, TMS8 and TMS10. This observation is in line with our previous proposals that specificity mutations define elements of selectivity filters or dynamic gates which allow or restrict the access of substrates to the *actual* binding site [11], [12], [19], [20].

In this work, we propose a structural model of UapA, through the implementation of a variety of computational methodologies. In addition, the construction and analysis of a number of rationally designed mutations was carried out, in order to gain further insight into the role of the various elements that constitute functional determinants of UapA. The group of residues experimentally characterized as critical for UapA function and specificity was identified and their role in substrate binding and transport was addressed in terms of structure as well as dynamics. The role of the functionally irreplaceable residues E356 and Q408 as the main interacting partners of the various UapA substrates was confirmed. A quantitative structure-activity relationship (SAR) model comprised by an extended set of UapA substrate analogues was constructed. The SAR model was in full agreement with our previous genetic and biochemical studies. Furthermore, advanced molecular simulations outlined a possible translocation mechanism for the physiological substrate by providing a trajectory-like displacement of xanthine across the protein and towards its cytoplasmic side. Possible selectivity gates at the outward and inward ends of the substrate translocation pathway are also proposed. We finally discuss the possible role of residues in the major binding site with respect to the specificity shift from nucleobases to ascorbate in members of the NAT family.

## Results and Discussion

### A UapA Structural Model

**Figure 1:**
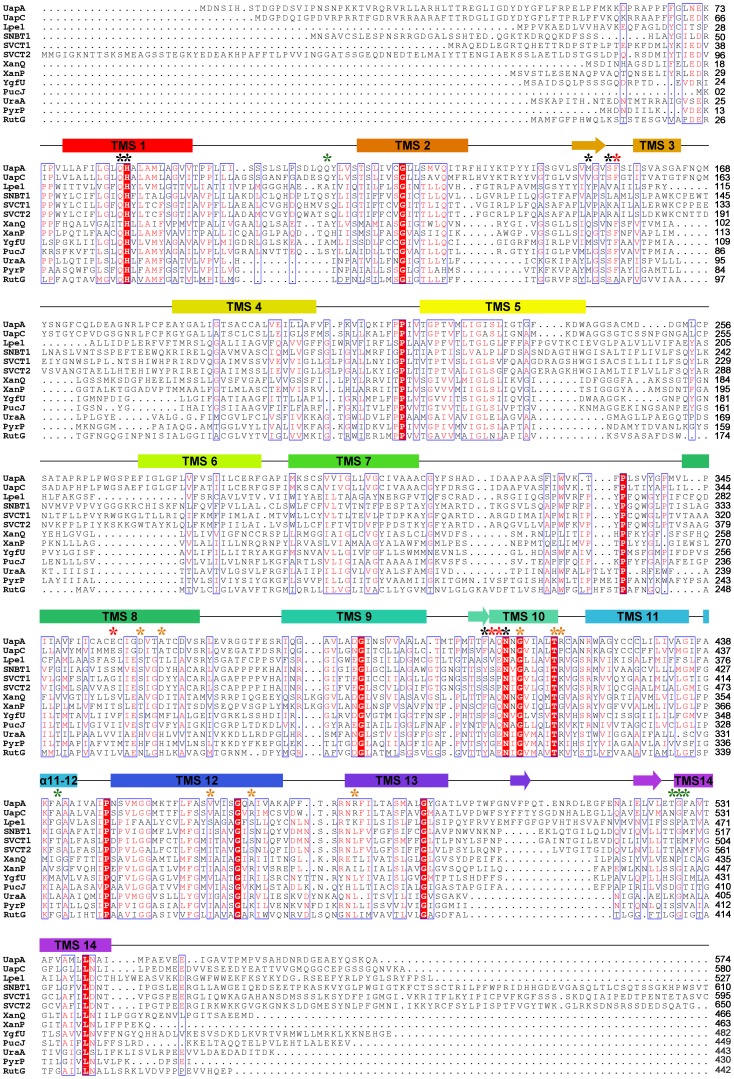
Multiple alignment of UapA, UraA and NAT homologues of know function and specificity, used for UapA modeled structure. Putative transmembrane segments (TMS) of UapA are denoted in colored cylinders. TMSs forming short β-sheets are shown with arrows. α stands for a-helical segments. Invariant and highly conserved amino acids are shaded in red and blue-lined boxes, respectively. Amino acids critical for function and specificity discussed in the text are highlighted with asterisks: red for residues of the substrate binding site, orange for those located in the substrate translocation pathway, green for aminoacids enlarging specificity and black for other important residues involved in dynamic interactions of TMSs. The listed NAT homologues include: UapA of *Aspergillus nidulans*, GI: 88984992; UapC of *Aspergillus nidulans*, GI: 790973; Lpe1 of *Zea mays*, GI: 162462794; SNBT1 of *Rattus norvegicus*, GI: 284010030; SVCT1 of *Homo sapiens*, GI: 6652824; SVCT2 of *Homo sapiens*, GI: 6048257; XanQ of *Escherichia coli*, GI: 161784262; XanP of *Escherichia coli*, GI: 84028014; YgfU of *Escherichia coli*, GI: 85675700; PucJ of *Bacillus subtilis*, GI: 16080296; UraA of *Escherichia coli*, GI: 187775829; PyrP of *Lactococcus lactis*, GI: 15673585 and RutG of *Escherichia coli*, GI:89107857.

The construction of a structural model of UapA became possible by the recent release of the crystal structure of UraA of *E. coli* [13], [21]. The two proteins share a rather moderate sequence similarity (23% identity, 41% positives), which is however adequate for sustaining a theoretical model of UapA, especially if combined with the plethora of the existing experimental data. The model built here was based on the multiple alignment of the NAT proteins of know function and specificity which was further modified manually so that it accommodates the correct version of UapA primary sequence (Uniprot accession number Q07307, replacing erroneous sequences CBF71770.1 and EAA57687.1) (Figure 1). Model building was performed using MODELLER software. This algorithm has been used recently with success for the norepinephrine transporter NET [23]. The loop refinement routine and a slow simulated annealing protocol for model refinement were implemented and 40 models were obtained. The structure with the optimal objective function was selected for further validation. As a first validation of the model, the structure with the best spatial restraints score was subjected to a 50 ns molecular dynamics run using Desmond software [24]. The system was prepared by embedding the protein in a POPC lipid bilayer and solvating the membrane by explicit water. The RMS deviation of the Cα-carbons of all Helices, from starting coordinates was monitored throughout the simulation and did not exceed 3.0 Å, thus indicating the stability of the theoretical model (Figure S1).

**Figure 2:**
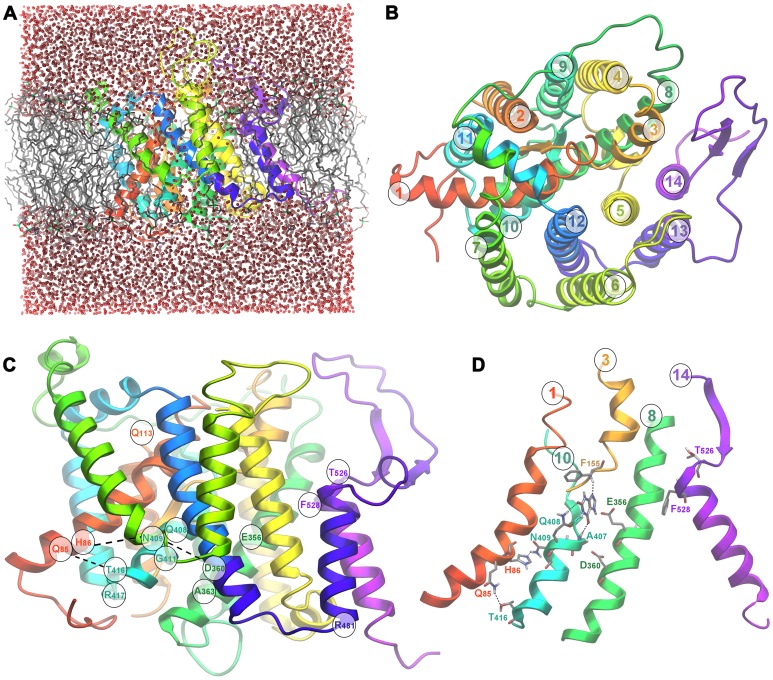
Theoretical structure of UapA. (A) Modeled 3D structure of the UapA validated with molecular dynamics using Desmond software. (B) Top view of UapA model, indicating core (TMS1–4, 8–11)/gate (TMS5–7, 12–14) domains and TMS numbering. (C) Side view of UapA structure showing the topology of residues selected as crucial for the function (Q85, H86, E356, A363, Q408, N409, G411, T416, R417) and specificity (Q113, R481, T526, F528) of UapA. (D) Detailed view of dynamic interactions between TMS1 (Q85, H86), TMS8 (D360) and TMS10 (N409, T416). TMS14 is also shown to highlight the position of residues T526 and F528, which are mostly critical for UapA specificity, in respect to all other important residues in TMS1, TMS8 and TMS10, involved in substrate binding and transport. In (a), (c) and (d) the upper part of the figure is outward-facing and the lower part is cytoplasmic-facing.

The overall 3D structure of the UapA model (Figure 2) corresponds to a cytoplasm-facing conformer made of 14 transmembrane segments (TMSs) that adopt a mostly helical secondary structure. The architecture of the transporter divides it in two distinct subdomains, the core which consists of TM segments 1–4 and 8–11 and the gate consisting of segments 5–7 and 12–14. The transmembrane helices are connected by large loops, the majority of which notably corresponds to lengthy insertions in the sequence alignment, thus posing an additional difficulty in obtaining an accurate conformational representation for this part of the transporter. The distribution of the ionized residues on the protein surface is fairly reasonable, as most of them are positioned either at the cytoplasmic and periplasmic sides or along the protein pore in the protein interior. Positive charges are mostly concentrated in the cytoplasm-facing loops.

### Genetic Support for the UapA Model

The topology of residues found to be crucial for UapA function through physiological, cellular and kinetic analyses of relevant mutants is indicated in Figures 1 and 2 and information on them is summarized in Table 1. The overall picture is that critical residues in terms of substrate binding and transport are positioned in good accordance with existing genetic and biochemical data. All residues so far identified as essential or critical for UapA function are located within TMSs 1, 3, 8 and 10 of the core domain. More specifically, the NAT signature motif Q/E/P-N-X-G-X_4_-T (Q^408^N^409^N^410^G^411^X_4_T^416^ R^417^ in UapA), which was proposed by functional studies to be an essential element for substrate recognition and transport, is located on TMS10 in a small 9-residue helix opposite TMS8, at the interface between the two protein subdomains (see Figure 2C). The importance of the NAT motif is dual. First, Q408 is directly involved in substrate binding, as strongly suggested by functional studies [9] its alignment with E290 which is a residue interacting with substrate in UraA [21], and from docking studies performed herein (see later). Second, N409, N410 and T416 seem to be involved in the stabilization of the protein tertiary intra-subdomain structure. More specifically, according to the model, a network of hydrogen bonds is formed between the side-chains of N409, H86, Q85 and T416 facilitating the interaction of TMS1 and TMS10, both belonging to the core subdomain (see Figure 2C, 2D). Experimental support for dynamic intramolecular interactions between these residues comes from the fact that substitution of any of the four residues confers cryosensitivity to UapA transport activity. Furthermore, most substitutions of these residues lead to a dramatic reduction in *V*_m_, but do not affect *K*_m_ values or the localization of UapA into the plasma membrane [9], [14]. A similar network is comprised by the side-chains of N410, T405 and D360, possibly strengthening the interaction between TMS8 and TMS10, which also belong to the protein core. Furthermore, the UapA model also revealed putative critical interactions of TMS1 with both TMS3 and TMS10. The importance of TMS3 (residues F155 and S154) and its interaction with TMS1 were genetically supported by functional studies of relevant mutants and by second-site suppressors of the H86D (TMS1) mutation, which are located in M151 (TMS3) [13]. Finally, residue E356 in UapA, which previous functional studies [11] and docking studies performed herein (see later) show that is the second major residue involved in direct interactions with xanthine (see below), aligns perfectly with E241 in the UraA structure, which is a residue shown directly to interact with the substrate. Significantly, an homology model of the structure of the XanQ permease in *E. coli* has also shown that functional mutations map in TMS1, TMS3, TMS8 and TMS10 [22]. Thus, functional studies in two evolutionary very distant homologous transporters, such as UapA and XanQ, validate the details of the crystal structure of UraA and the modeled structures of UapA and XanQ, especially as far as it concerns the substrate binding site.

**Table 1:** Summary of residues critical for UapA function and specificity.

Allele	Location	CoreDomain	GateDomain	Effect on transportcapacity^1^	Major Substrate binding site	Trajectory (t)or Gate (g)	Enlargedspecificity	Critical polar interactions
Q85	TMS1	+	−	+	−	−	−	T416
H86	TMS1	+	−	+	−	−	−	N409
Q113	TMS1–2 loop	+	−	+ or −	−	−	yes	−
M151	B3/TMS3	+	−	+/−	−	−	−	−
S154	TMS3	+	−	+/−	−	−	−	−
F155	TMS3	+	−	−	yes^2^	−	−	−
E356	TMS8	+	−	+	yes	−	−	−
D360	TMS8	+	−	+	−	t	−	T405, N410
A363	TMS8	+	−	−	−	t	−	−
F406	B10/TMS10	+	−	−	−	−	yes	
A407	TMS10	+	−	+	yes^2^	−	−	−
Q408	TMS10	+	−	+	yes	−	yes^3^	−
N409	TMS10	+	−	+	−	−	−	H86
G411	TMS10	+	−	+ or −	−	t	−	−
T416	TMS10	+	−	−	−	t	−	Q85
R417	TMS10	+	−	−	−	t	−	−
A441	TMS11–12 loop	−	+	−	−	−	yes	−
V463	TMS12	−	+	−	−	t	yes	−
A469	TMS12	−	+	−	−	t	yes	−
R481	TMS13	−	+	−	−	−	yes	−
T526	TMS14	−	+	−	−	g	yes	−
G527	TMS14	−	+	+	−	g	yes	−
F528	TMS14	−	+	−	−	g	yes	−

TMS: transmembrane segment.

B: beta sheet conformation within the TMS.

1 “−”: no major effect on Vmax >50%, “+”: major effect on Vmax <10%, “+/−”: Vmax <30% and “+ or −“: depending on specific substitution.

2 Evidence for involvement in substrate binding through peptide backbone interactions, as shown by docking. Consequently mutations with relatively small amino acids do not have an effect.

3 Q408E, confers ability to bind hypoxanthine and guanine, but does not lead to their transport. Q408 in combination with gate mutations leads to high-medium affinity binding and transporter of all purines and uracil.

Interestingly, in UapA, elements distinct from the binding site, located in the C-terminal part of the protein (TMS12–TMS14) and in the TMS1–2 loop (see Figure 2C), were shown to control substrate specificity, thus supporting the idea that NATs consist of two topologically and functionally distinct structural folds, the core and the gate domain, as this was proposed for the UraA structure [21]. This observation also formed the basis of our previous proposal which stated that specificity mutations define distinct selectivity filters or dynamic gates which allow or restrict the access of substrates to the actual binding site (see also later). Noteworthy, UapA and XanQ/UraA have significant structural differences in their gating domains, which is reflected in genetic and functional differences [11], [20], [25]. This is highlighted by mutations in TMS14 concerning residues T526 and F528 in UapA, which correspond to N430 and Ile432 in XanQ. In particular, while T526 and F528 mutations enlarge dramatically the specificity of UapA, the analogous mutations in XanQ affect mostly the transport kinetics in respect to the physiological substrate xanthine and much less the specificity for certain xanthine analogues with bulky substitutions. This observation suggests that in the course of evolution UapA has acquired a more flexible gating domain, a hypothesis in line with a significant longer TMS13–TMS14 in UapA compared to XanQ.

### Substrate Docking Leads to a Model for Xanthine-UapA Interactions

A major objective of the present study was the elucidation of the recognition process between UapA and its physiological substrates and their subsequent translocation along the transporter pore. To this respect two objectives were pursued. The first was the determination of the role played by residues which have been identified through genetic studies as critical for interacting with the physiological substrate. The second was the construction of a structure-activity relationship hypothesis based on those results, which could in turn facilitate the design of compounds that by competing with the physiological substrates could act as inhibitors with potential medical importance. To approach the issues of substrate recognition and translocation, a model of the interaction between UapA and xanthine was created using docking-scoring calculations and the structure of UapA as derived from homology. Docking calculations were performed using two distinct docking protocols, a protocol based on the mixed low-mode/Monte Carlo sampling algorithm for flexible docking and the Induced Fit Docking protocol (IFD) as introduced by Schrodinger 2011 Suite of programs. The IFD protocol is based on an iterative implementation of Glide algorithm for rigid docking and Prime algorithm for protein refinement, resulting in an improved simulation of binding in terms of protein flexibility. This allows for a highly efficient and sophisticated compromise of docking speed and binding accuracy. Furthermore, since Prime is a modeling tool especially developed for refinement of protein structures derived by homology, its implementation as part of the IFD protocol was considered in the case of UapA as promising since it represented an approach complementary to the classical low-mode/Monte Carlo where the protein is modeled as flexible. An additional issue that was addressed concerned the tautomerism of xanthine. In neutral pH xanthine adopts two dominant, almost equally populated tautomeric states [26] which however introduce a key difference to the hydrogen bond properties of each isomer. The different protonation states of N7 and N9 and the corresponding tautomers are denoted as Xan7H and Xan9H respectively.

**Figure 3:**
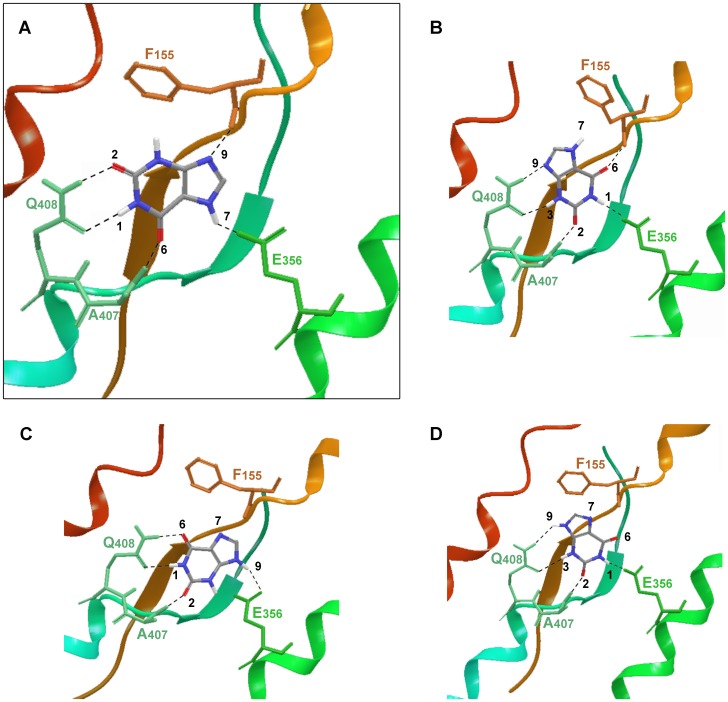
UapA – substrate interactions. Schematic representation of the four different docking poses (A–D) of xanthine-UapA interactions (models 1–4 accordingly). Poses (A) and (B) show the modeled UapA-Xan7H complex and poses (C) and (D) the modeled UapA-Xan9H complex. The most favoured model is shown framed and in bigger scale (A). This model was supported by docking using mixed low-mode/Monte Carlo sampling algorithm for flexible docking and the Induced Fit Docking protocol (IFD), as well as, SAR.

Docking results were fairly consistent with genetic data however relatively inconclusive in suggesting a unique binding mode for xanthine. Two different docking poses were obtained as lowest energy structures for each tautomer, poses 3A and 3B for the UapA-Xan7H complex and poses 3C and 3D for the UapA-Xan9H complex (Figure 3). In pose 3A Xan7 is stabilized in the protein substrate binding domain by 5 hydrogen bonds. In that pose Q408 plays a key role in binding, in good agreement with data suggesting a direct contribution of that residue to substrate recognition [9], [11]. The aforementioned hydrogen bond which is formed between Q408 side chain amide and xanthine is also present in pose 3C describing the UapA-Xan9 interaction. Yet, while in pose 3A the binding partners of Q408 are the NH at position 1 and the carbonyl at position 2 of Xan7H purine ring, in pose 3C the respective interaction sites are NH at position 1 and the carbonyl at position 6 of Xan9H. Genetic data have denoted the carboxylate of E356 as an essential element in protein recognition [11]. Docking results were in accordance with these data as it was shown that this residue interacts through a hydrogen bond with the NH at either position 7 of Xan7H or position 9 of Xan9H and possibly influences the orientation of the ligand inside the protein binding pocket (Figure 3). Two additional interactions further contribute to the stabilization of the complexes, the interaction between the backbone NH of A407 and either 6-carbonyl (Xan7H) or 2-carbonyl (Xan9H) and the interaction between the backbone carbonyl of F155 and either N9 of Xan7H or N7 of Xan9H. In pose 3B of the UapA-Xan7 complex, the substrate is also anchored through 5 hydrogen bonds. In that geometry Q408 interacts with the N3H and N9 of the purine while E356 interacts with NH1. Finally, in pose 3D the UapA-Xan9H is stabilized by 4 hydrogen bonds, where Q408 contributes only one hydrogen bond while E356 interacts with the N1H of xanthine. Interestingly, the interaction between A407 and F155 backbone and the substrate is conserved in all four poses with small variations on the xanthine interaction partners. An additional favourable interaction which was commonly present in all four binding orientations was the π-π stacking of the electron-rich purine system between the side-chains of F406 and F460.

To further explore the conformational space of the UapA-xanthine complex and evaluate the convergence of the IFD protocol, fully flexible docking calculations were undertaken using the low mode/Monte Carlo sampling method. The results from the IFD calculations were used as starting structures. The consistency between the two methodologies was fair. In the case of tautomer Xan7H, the global minimum structure of the UapA complex was identical to pose 3A originating from IFD calculations. However, the global minimum structure of Xan9H inside the binding pocket was not close to poses derived from IFD calculations, and lacked specific interactions with the protein. Thus, while docking calculations have provided a rich insight to the recognition process which was in full consistency with genetic data, they were inconclusive in determining the dominant binding geometry out of the four possible Xan orientations and/or protonations.

### SAR Confirms the Mode of Xanthine Binding to UapA

**Figure 4:**
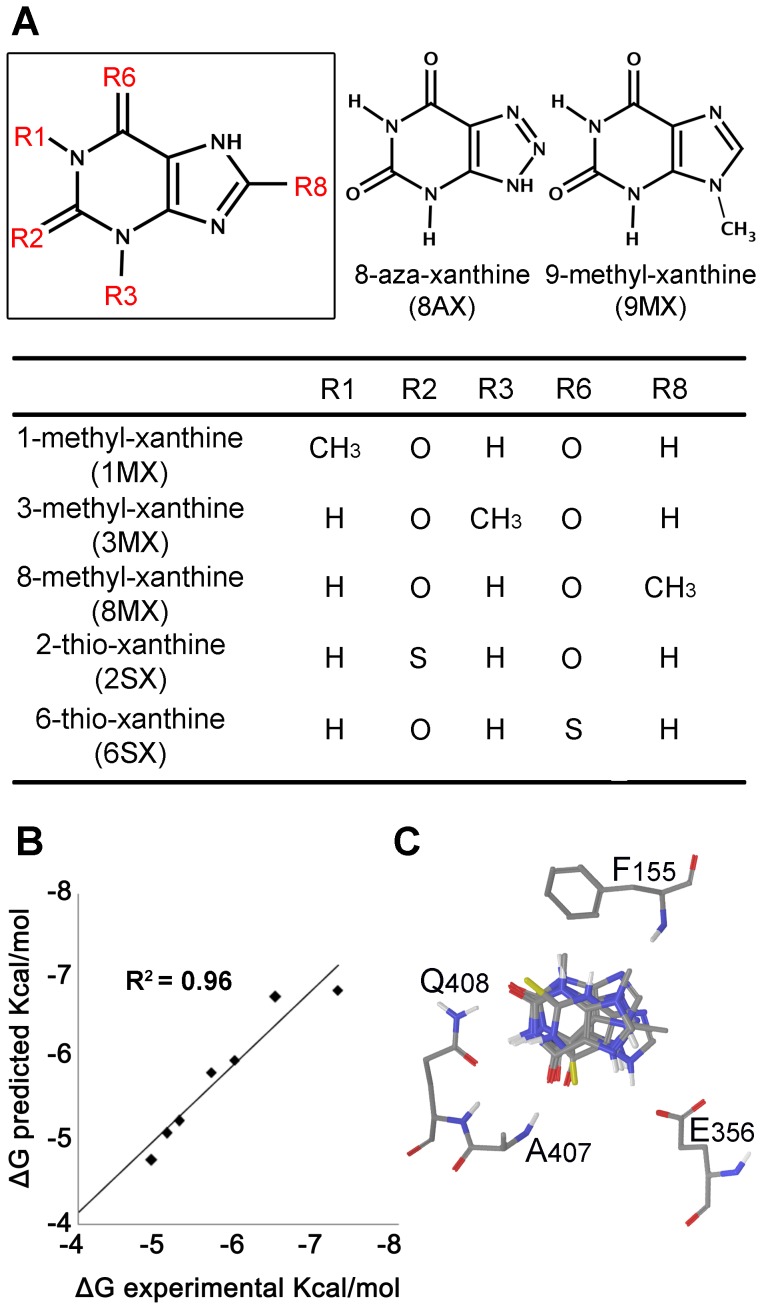
Structure Activity Relationship (SAR) model for the interaction of UapA with xanthine analogues. (A) Structures of XAN analogues used for model creation. (B) Predicted VS Experimental ΔG_binding_. (C) Superposition of XAN analogues inside binding domain of UapA as proposed by final model.

To further check the consistency of the four different binding modes of Xan with all existing experimental data, we attempt the creation of quantitative Structure Activity Relationship (SAR) models by considering a small set of xanthine analogues with known free energy of binding (Figure 4A). For that purpose, the iterative docking-scoring methodology of PrGen 2.1 software was used [27]. Theoretical binding affinities (E_binding_) were estimated by evaluating ligand-receptor interaction energies, ligand desolvation energies and changes in both ligand-internal energy and ligand internal entropy upon receptor binding (see Materials and methods). Calculated free energies ΔG°_pred_ were then obtained by linear regression between experimental free energy ΔG°_exp_ and E_binding_. A training set of seven xanthine analogues was used (XAN, 2SX, 6SX, 3MX, 8MX, 9MX, 8AX) and four different models were created by superimposing all ligands to each of the four poses of xanthine, using for each analogue the appropriate tautomer. Xanthine analogues showing a very low binding affinity, such as 1-methylxanthine and hypoxanthine were excluded from the training set, but retained as test set. The quality of each model was evaluated by the coefficient of determination r^2^ for the correlation between experimental and predicted ΔG°and the degree of deviation from Xan orientation. While models 3A and 3C demonstrated a satisfactory correlation (r^2^ of 0.958 and 0.964 respectively), a poor r^2^ was determined for models 3B and 3D (0.567 and 0.473).

In model 3A the good correlation (Figure 4B) was accompanied by docking poses (Figure 4C) of the different analogues that were in agreement with the pose of xanthine, while a repositioning of all analogues with respect to starting pose was evident in model 3B. It is considered that the high degree of structural similarity between Xan and the selected analogues introduces the necessity of an equally high degree of alignment of the purine scaffold within the binding cavity. Thus, model 3A was selected as the consensus of all three approaches utilized. Model 3A was further validated by calculating the binding energy of the test set. Calculated binding energies using Prgen software showed that their binding affinity was higher than −4.3 Kcal/mol, which is in good agreement with experimental values.

### Functional Studies Validate the Proposed Xanthine-UapA Interaction

Model 3A could very well explain the substrate specificity profile of UapA. 3-methylxanthine (3MX), which is a good ligand, is positioned very similar to xanthine (XAN) (Figure S2A). The methyl group is placed near the phenyl group of F155 forming weak Van der Waals interactions. 8-methylxanthine (8MX) is a moderate binder, showing steric hindrance with the methyl group of the side chain of T404 and the carboxyl group of E356 lowering the binding affinity compared to XAN. The hydrogen bond between N7-H and COOH_E356_ still exists but is weaker (Figure S2B). 9-methylxanthine (9MX) (Figure S2C) shows moderate binding affinity too, as the methyl group is placed close to the NH group of the backbone of F155 disrupting the N9-NH_F155_ H-bond. 1-methylxanthine (1MX), which is a non-binder, is considerably displaced, lacking interaction with Q408 and E356 (Figure S2D). 2-thioxanthine (2SX) is better binder compared to 6-thioxanthine (6SX) (Figure S2E–S2F). The C = S bond is longer than C = O, displacing substrate 2SX towards E356 while 6SX is displaced in opposite direction, towards F155. Thus, 2SX forms most of the interactions found in XAN, (however the sulphur-containing hydrogen bond is weaker compared to the oxygen one [28]), while 6SX lacks H-bond with E356. 8-azaxanthine (8AX) although is positioned identical to XAN (Figure S2G), is a weak binder, probably because of the stereoelectronic properties of the N-N = N group, preventing the way in and/or the translocation through the transporter. Finally hypoxanthine (HX) cannot form hydrogen bond with Q408 and E356 on the same time and thus is totally displaced inside the binding cavity compared to XAN (Figure S2H). Additionally docking calculations were performed for purines not recognized by the wild-type UapA, such as guanine and adenine taking into account their different tautomeric states. In both cases the Q408 amide failed to form bidentate hydrogen bond with the substrate, resulting in fewer H bond interactions compared to XAN (Figure S2I–S2J).

In line with this model, residues Q408 and E356 are absolutely necessary for substrate binding and transport (even the most conserved substitutions Q408E and E356D lead to dramatic loss of transport activity), while residues A407 and F155 can be functionally replaced [9], [11], [13]. Further evidence for the direct involvement of Q408 and E356 in substrate binding comes from the fact that the mutation Q408 confers the ability for binding novel substrates (hypoxanthine and guanine) and mutation E356D leads to 18-fold increased affinity for xanthine but abolishes transport. This last finding should be emphasized as it provides indications which might sustain a hypothesis about the role of E356 not only to direct substrate binding but to the dynamics of the inward-outward transporter transition, as well. Flexible docking calculations of XAN to the E356D-UapA, clearly demonstrated that although of minor influence in terms of physicochemical properties and interaction profile, the mutation of glutamate to an aspartate 2-thioxanthine is however critical with respect to the directional flexibility of the side chain involved. The shorter side chain of D356 poses a serious limitation to the conformational space accessible by the carboxylate functionality compared to the wild type protein. This constraint acts synergistically with the highly ordered assembly of the three residues that are engaged in the interaction with the pyrimidine ring of xanthine. As a result, the concurrent and finely tuned anchoring of xanthine to all four interaction partners F155, E356, A407 and Q408 through H-bonds is no longer feasible as a consequence of the reduced conformational flexibility of the latter. It can be speculated that a failure in the formation of a stable and optimally equilibrated complex could negatively affect the energetics of the conformational shift and thus lead to perturbed transporter functionality as was experimentally determined for this mutant. A mechanistic explanation for that perturbation might involve the interaction of E356 with residues located across the pore, its role as a mediator of the sliding of xanthine towards D360 and most importantly its possible functionality at the proton symport cascade. As a summary, we speculate that the shorter side-chain of D356 reduces its capacity to interact with the substrate, as clearly shown in docking results where no acceptable pose of xanthine directly bound to D356 was found. That in turn might negatively influence the transition from an outward-to an inward-facing conformation necessary for transport catalysis [29], [30]. Indirect support for this speculation comes from the fact purified UapA-E356D protein is significantly more stable than the wild-type protein [31].

### Identification and Genetic Support of a Cytoplasm-facing Substrate Trajectory

**Figure 5:**
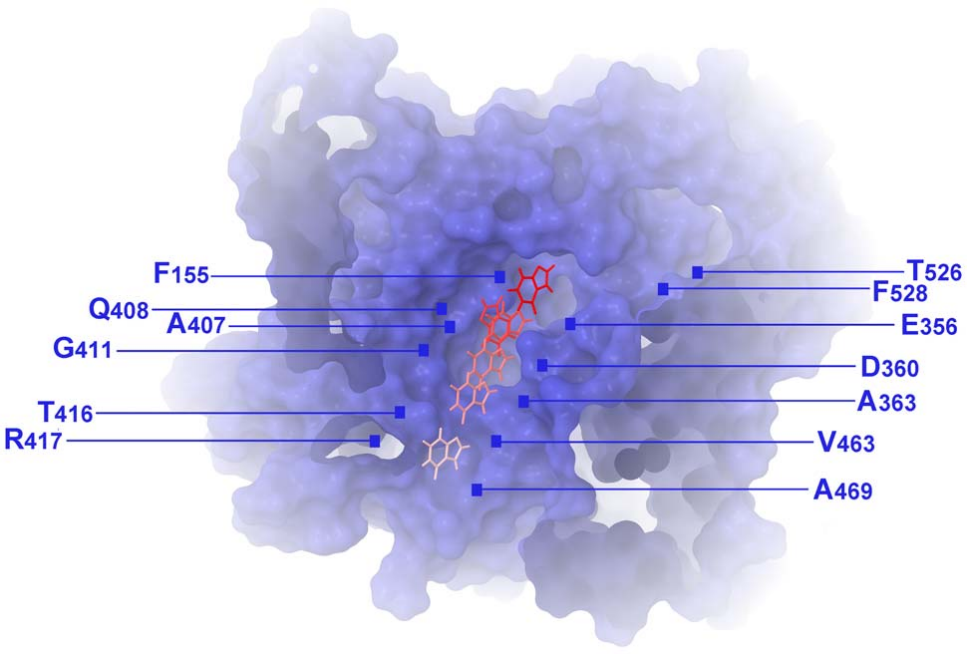
A xanthine translocation pathway in the cytoplasm-facing UapA model. Residues F155, Q408, E356 and A407 define the major substrate binding site, whereas T526 and F528 indicate a putative outward-facing gate (see text).

Apart from predicting the binding mode of xanthine, another challenging aspect when studying a transporter is to predict the trajectory path of the substrate. Flexible docking calculations were chosen for this study, since they can randomly position the ligand inside the binding pocket using specific rotational and translational algorithm. The conformational space of the complex was extensively sampled by using the Monte Carlo and Low Mode conformational search algorithm. This method has proven highly efficient in sampling similar systems and it is considered as a robust technique [32]. Starting from the binding pocket previously originated xanthine was placed about 6 and 12 Å along the channel formed by TMSs 8, 10 and 12 on both directions producing 5 initial structures for docking calculations. 5000 steps of Monte Carlo/Low Mode were produced for each run, followed by energy minimization. During Monte Carlo perturbation the ligand was free to move along the x, y, z axes from 0 to 5 Å and simultaneous free rotation. The lowest energetically structures obtained are depicted on Figure 5 providing a theoretical pathway of the ligand before and after the binding pocket.

The proposed substrate translocation pathway starts from the centrally located major substrate binding site (residues F155, E356, A407 and Q408) and is followed by subsequent poses of xanthine docking towards the cytoplasmic face of the transporter, close to residues D360, A363, G411, T416, R417, V463 and A469 (Figure 5).

**Figure 6:**
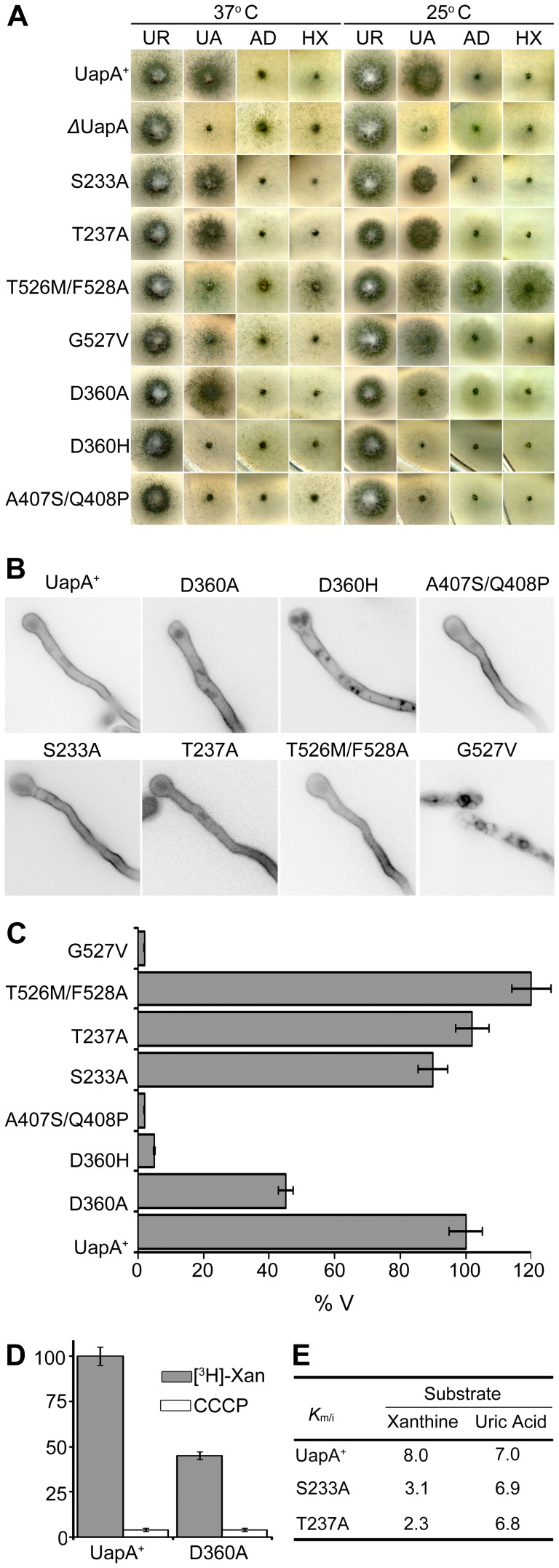
Functional analysis of new UapA mutations. (A) Growth tests on purines as sole nitrogen sources at 25 and 37°C. UA indicates uric acid, AD is adenine, HX is hypoxanthine. As a control, growth on urea is also shown (UR). Positive (UapA^+^) and negative (*Δ*UapA) isogenic control strains are also shown. (B) Epifluorescence microscopy showing *in vivo* subcellular expression of UapA-GFP mutant alleles and a wild-type control (UapA^+^). (C) Comparative initial uptake rates of ^3^H-radiolabled xanthine in UapA mutant alleles and a wt control. 100% is the transport rate in the wt (UapA^+^). (D) *K*_m_ values for functional UapA mutants and wt (UapA^+^). For details see Materials and Methods.

D360, which is a very well conserved residue in NATs, has not been mutated before. However, the equivalent Asp in XanQ was shown to be absolutely necessary for xanthine transport [22] and in UraA it corresponds to H245, a residue speculatively proposed to be important for a proton-coupled mechanism of transport of Uracil [21]. We mutated Asp360 to Ala and His. D360H scored as a total loss-of-function mutation (Figure 6A, 6C) and despite being localized in the plasma membrane showed increased levels of vacuolar turnover (Figure 6B). D360A was relatively stably localized in the plasma membrane (Figure 6B), but conserved low transport activity, mostly at 37°C (Figure 6A, 6C). Interestingly, the low transport activity of UapA-D360A was dependent on the plasma membrane proton gradient and pH, similar to the wild-type allele (Figure 6D). Furthermore, D360A showed substrate affinity and specificity profiles very similar to the wild-type protein (see Figure 6A and results not shown). These results contradicts the participation of D360 as a residue essential for the binding and symport of H^+^ and rather supports an indirect role in substrate translocation, possibly through its interactions with N410 and T405, as shown earlier (see Figure 2).

A363 and G411 have been shown to be critical residues for transport [9], [33]. Noteworthy, specific substitutions of G411 either immobilize UapA (G411V) [31], or increase 2-fold its apparent *V* (G411A, G411V) [9], suggesting that G411 is a key dynamic element in movements associated with UapA-mediated transport. Residue R417 has been shown to be important specifically for increasing uric acid binding affinity [10]. In line with that, mutation R417G reduces dramatically uric acid binding but conserves high affinity for xanthine.

The other two residues, V463 and A469, do not seem to be important for substrate transport *per se*, but specific substitutions of them affect significantly UapA specificity [12]. Most interestingly, none of the above residues is critical for protein turnover or for substrate binding, as shown by relevant mutations. Thus, all elements of the proposed substrate trajectory are associated with mutations that either affect transport rates (apparent *V* values) or substrate specificity. This observation is in excellent agreement with residues lining a cytoplasm-facing trajectory downstream from the major substrate binding site.

### Genetic and Structural Support for a Dynamic Outward-facing Gate Critical for UapA Specificity

Among the most prominent, genetically selected, specificity mutations are substitutions of T526 and F528 with aliphatic or polar amino acid residues (Met and Leu for T526; Ala, Ser, Thr for F528). These substitutions do not affect the kinetic and specificity profile of UapA for its natural substrates (uric acid and xanthine) but confer UapA-mediated low affinity uptake of other purines and purine analogues with bulky substitutions [11], [20]. Based on these finding we have proposed that these two residues act as elements of a molecular filter or a dynamic gate which selects which purines can have access to the major substrate binding site, and in turn, substitutions of T526 and F528 loosen the selectivity of this gate. In the UapA model built herein, T526 and F528 are located in the outward-facing edge of TMS14, ideally positioned for defining the entrance of substrates in a trajectory leading to the major binding site. Does this putative outward-facing gate also act as a secondary substrate binding site? Although most evidence supports the existence of a single major substrate binding site positioned in a central cavity of all transporter studied, the existence of secondary binding sites in outward and inward faces of transporters is a recent and strongly debatable issue [34]–[41].

**Figure 7:**
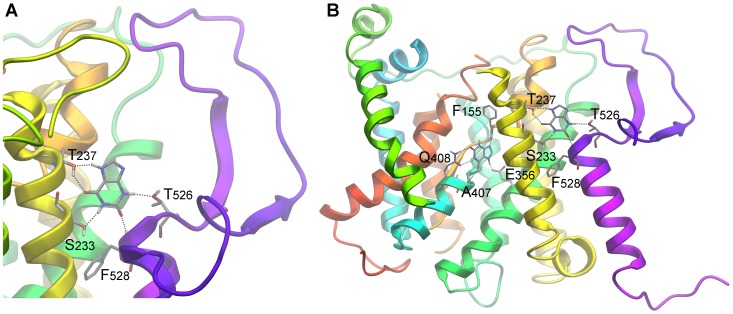
A putative xanthine secondary docking pose at the extra-cytoplasmic side of TMS14. (A)Detailed view and (B) its relative position to the primary binding site.

To test the possible implication of residues T526 and F528 as elements of an outward-facing gate and/or a putative secondary substrate binding site, we performed flexible docking calculations of xanthine utilizing wide sampling. Our results indicated a particular binding geometry at a distance from the major substrate binding domain, which might serve as an individual outward-facing recognition spot and which includes residues T526 and F528 (Figure 7A, 7B). More specifically, a small ensemble of poses with favorable geometries is found to occupy a cavity formed at the boundary between the extracellular and transmembrane regions of the protein. In this area, a well defined cleft is formed between the gate subdomain of the transporter and protruding helices TMS13 and TMS14. The purine is stabilized there by hydrogen bonds accommodated by S233, T237, T526 and F528. It should be mentioned that from a topological perspective that cavity is simultaneously the most easily accessible from the solvent and still in very close proximity to the major binding site (distance between E356 and F528 is only 12 Å). Thus it might be considered that the major and a secondary substrate binding site are interconnected, as a linking path would be easily assumed by hypothesizing slight movements and rolling of helices TMS4 and TMS7. This proximity is further supported biochemically in XanQ where substrate binding protects the alkylation of cysteine residues genetically positioned in TMS14 [25].

To further confirm the existence of this outward-facing gate and test whether it also functions as a secondary substrate docking site, we constructed and analyzed mutations concerning S233 and T237 and G527, which have not been mutated before. We also constructed and analyzed the double substitution T526M/F528A. According to the docking result, substitutions of S233 and T237 would, in principle, enlarge the specificity profile of UapA, similar to substitutions in T526 and F528, whereas substitutions G527V would also affect the local architecture and thus loosen the specificity of UapA. The double substitution T526M/F528A might also further loosen UapA specificity. Results for the analysis of these mutations are shown in Figure 6. Ala substitutions of S233 and T237 did not affect at all the plasma membrane localization, the turnover or the transport kinetic and specificity profile of UapA, strongly suggesting that these residues are not part of the outward-facing gate or of a secondary binding site. In contrast, G527V scored as a loss-of-function mutation, especially at 37°C (Figure 6A, 6C), apparently due to protein instability and vacuolar degradation (Figure 6B), whereas T526M/F528A showed, temperature-dependent, enlarged UapA specificity, leading to UapA-mediated growth on adenine or hypoxanthine transport (Figure 6A), strongly supporting the role of the relevant residues in the functioning of dynamic selectivity gate. In summary, the analysis of the new mutations further supports the role of residues T526, G527 and F528 as elements of an outward-facing dynamic gate controlling substrate specificity, but fail to provide genetic support for the presence of a secondary substrate binding in this gate.

Another outward-facing residue that affects dramatically UapA specificity is Q113 located in the loop between TMS1 and TMS2 (see Figures 1 and 2). A specific substitution, Q113L, enlarges UapA specificity similar to mutations concerning T526 and F528. We did not obtain a docking pose of xanthine close to Q133, which in fact seems very distant from the both the major substrate binding site and residues T526 and F528. We do not understand at present how this residue might affect the specificity of UapA, but it is not however uncommon in transmembrane proteins that a mutation might have domino effect on activity.

### A Possible Inward-facing Gate?

Besides Q113, T526 and F528, all other randomly selected mutations which have a prominent effect UapA specificity concern residue R481 [12]. Substitution of R481 with aliphatic residues enlarges the specificity of UapA similar to mutations in Q113, T526 and F528. This residue is located at the border of loop TMS12–TMS13 with TMS13 (see Figure 1 and 2). We did not obtain a docking pose of xanthine at this site, which is distantly located form the major substrate binding site. How is critical for the specificity of UapA for uric acid or xanthine is not, at present understood. It might be through a domino effect on the outward-facing gate or it could define an element of a dynamic inward-facing gate. We favor the second hypothesis based on two observations. First, deletion of R481 or a 2 amino acid insertion (Ala-Gly) immediately upstream from R481 lead to increased, temperature-dependent, UapA instability and vacuolar turnover, usually obtained with mutations in dynamic elements of the transporter [12]. Second, double mutants including R481 and substitutions in T526 or F528, further loosen the specificity of UapA, showing that there is an additive effect of outward-and inward-positioned mutations, which can be more easily rationalized if two independent selectivity gates operate at both sides UapA. Furthermore, despite the fact that R481 is topologically distant from the cytoplasmic end of the substrate translocation trajectory defined herein, which is close to R417 (see Figure 5), it should be taken into account that the UapA model built here is based on a static inward-facing conformation of UraA and consequently it should not be excluded that in a putative outward-facing conformation of UapA, loop TMS12–TMS13 and R481 are proximal to the cytoplasmic end of the substrate translocation trajectory.

### In Search of a Structural Rationale for the Evolution of Novel Specificities in the NAT Family

**Figure 8:**
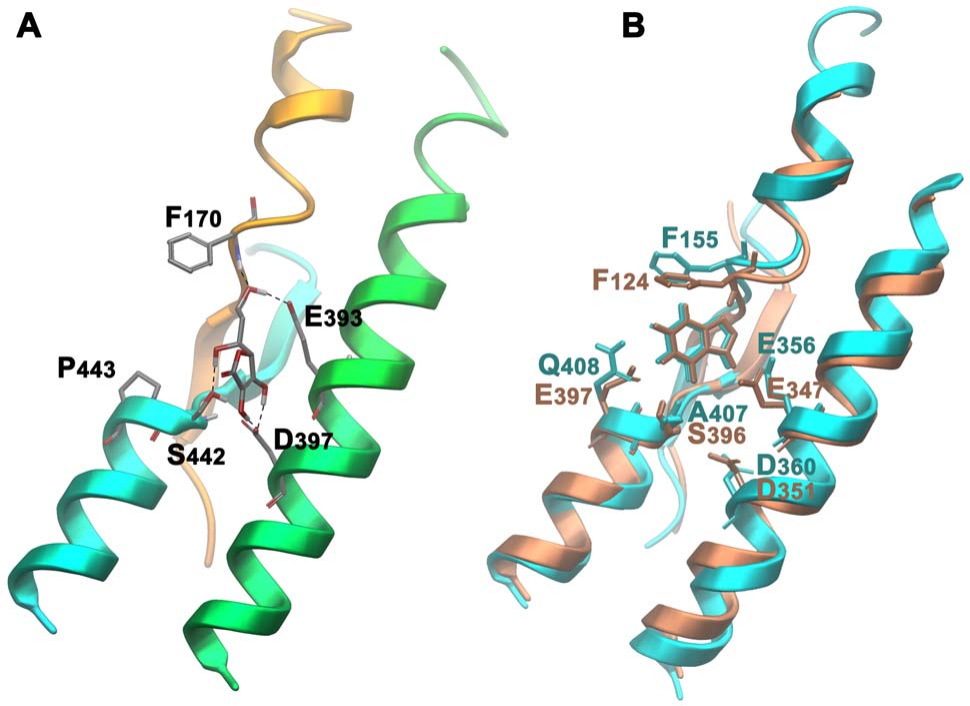
Interactions of NAT proteins with specific substrates. (A) Docking pose of L-ascorbate in SVCT-2 and (B) xanthine on superimposed rSNBT1 (in pink) and UapA (in blue).

In primates NAT members are specific for L-ascorbate/Na^+^ rather than nucleobases/H^+^. Other mammals have both NAT versions, specific for either L-ascorbate or nucleobases [1]. We modeled and performed docking studies with the rat nucleobase transporter rSNBT1 and the human L-ascorbate transporter SVCT2. Results, shown in Figure 8, demonstrate that L-ascorbate and xanthine dock specifically in a centrally located binding site in SVCT2 and rSNBT1 respectively, but not *vice versa*. The amino acid residues involved in substrate interactions in rSNBT-1 are identical or highly conserved compared to those identified in UapA (F124, E347, E397 and S396 in rSNBT1 corresponding to F155, E356, Q408 and A407 in UapA. In contrast, in SVCT2, which lacks the critical substrate binding Gln/Glu residue found in nucleobase-specific NATs (Q408 in UapA, E397 in rSNBT1), binds ascorbate using F170, S442, E393 and D397, residues that correspond to F155, A497, E356 and D360 in UapA. Thus, it is clear that the ‘replacement’ of a Gln/Glu by a Pro residue in the NAT motif, located in TMS10, is a crucial difference for the shift in specificity in this family of transporters.

To test whether this difference is sufficient to shift the specificity of UapA from purines to L-ascorbate we constructed mutant UapA-A407S/Q408P, and tested its expression, stability and transport profile in respect to purines and L-ascorbate. Results included in Figure 6 show that the double mutation does not affect the expression of a GFP-tagged version of this UapA allele to the plasma membrane, a very strong indication that the overall folding of the transporter is not affected. This mutant, however, has lost any detectable capacity of purine uptake and has not acquired identifiable uptake of L-ascorbate. This result strongly suggests that evolution of novel specificities within a transporter family is not simply a result of local changes in the major substrate binding site, but might also depend on other elements, such as outward-or inward-facing gates and molecular filters.

### Concluding Remarks

The present work presents a theoretical UapA structural model, which reveals a number of important aspects concerning how this transporter selects and transports its substrates. Results derived from different docking methodologies in conjunction with SAR modeling, were in very good agreement, thus proposing a highly consistent model concerning UapA substrate recognition. Obviously all structural models should be treated with great caution when used to speculate on function. However, UapA presents a unique case where a plethora of mutations, including randomly selected mutations, are available and have been used to understand function without knowing the structure of the transporter. Much to our satisfaction, our previous and present genetic and biochemical data fully support the structural data proposed in this work, and allowed us to speculate on a solid experimental ground.

Furthermore, our docking approaches are not only in excellent agreement with the *in vivo* specificity profile of UapA, but also provided a rationale for the difference in substrate specificity between the rat and the human NAT homologues, the former being specific for nucleobases and the latter for L-ascorbate. We have previously proposed that the presence of a Gln or Glu residue in the NAT motif (Q408 in UapA) is a molecular signature for predicting whether a NAT protein is specific for nucleobases rather than L-ascorbate. In L-ascorbate transporters Gln/Glu is replaced by a Pro residue. Here we provide strong mutational and structural evidence for this observation.

This work reinforces the novel concept of the existence of dynamic gates or molecular selectivity filters in specific families of transporters [2], [42], [43]. The existence of filters or gates can be easily reconciled with the generally accepted rocker-switch mechanism of alternating outward-and inward-facing conformational states in transporters underlying their functioning [30]. Gating, which introduces occluded and open intermediates in the outward-and inward-facing conformers, might have evolved to add extra specificity or to prevent leakage of substrates in the wrong direction [44].

Our findings further show that specificity of NAT homologues belonging to evolutionary distant groups, such as fungi and metazoa might not solely be determined from specific interactions within a major, centrally located, substrate binding site. When we genetically constructed a UapA substrate binding site mimicking that of the human ascorbate transporter SVCT2, we obtained an apparently inactive UapA transporter. This strongly suggests that the mutational barrier underlying the specificity shift between UapA and SVCT2 extends beyond changes in the substrate binding site and probably includes changes in dynamic elements of these transporters, including gates and molecular filters, as those described herein. This observation should be critical in future efforts to use NAT transporters as specific gateways for developing targeted antimicrobials, but also for rationally designing *in vitro* evolution approaches for understanding how transporters work.

## Materials and Methods

### Homology Modeling

Homology model building was performed using MODELLER v.9.8 software [45].

### Protein Preparation

The protein was prepared for the docking calculations using the Protein Preparation Workflow (Schrödinger Suite 2011 Protein Preparation Wizard) implemented in Schödinger suite and accessible from within the Maestro program (Maestro, version 9.2, Schrödinger, LLC, New York, NY, 2011). Briefly, the hydrogen atoms were added and the orientation of hydroxyl groups, Asn, Gln, and the protonation state of His were optimized to maximize hydrogen bonding. Finally, the ligand−protein complex was refined with a restrained minimization performed by Impref utility, which is based on the Impact molecular mechanics engine (Impact version 5.7, Schrödinger, LLC, New York, NY, 2011) and the OPLS2001 force field, setting a max rmsd of 0.30. Ligand preparation for docking was performed with LigPrep (LigPrep, version 2.5, Schrödinger, LLC, New York, NY, 2011) application which consists of a series of steps that perform conversions, apply corrections to the structure, generate ionization states and tautomers, and optimize the geometries.

### Molecular Dynamic Simulations

For the MD simulations Desmond v.3 software was implemented (Desmond Molecular Dynamics System, version 3.0, D. E. Shaw Research, New York, NY) [24]. The system was prepared by embedding the protein in a POPC lipid bilayer, solvating the membrane by TIP4P explicit water, neutralizing with counterions and adding 150 mM salt and subsequently following the stepwise equilibration protocol as developed by Desmond for membrane proteins. The 50 ns simulation was performed in the NPγT ensemble with Langevin thermostat and barostat and semi isotropic pressure restraints. All molecular dynamic simulations were run on Cy-tera HPC facility (http://www.linksceem.eu/ls2/).

### Induced Fit Docking

Molecular docking was performed using the Induced Fit Docking (IFD) protocol [46] (Schrödinger Suite 2011 Induced Fit Docking protocol), which is intended to circumvent the inflexible binding site and accounts for the side chain or backbone movements, or both, upon ligand binding. In the first stage of the IFD protocol, softened-potential docking step, 20 poses per ligand were retained. In the second step, for each docking pose, a full cycle of protein refinement was performed, with Prime 1.6 (Prime, version 3.0, Schrödinger, LLC, New York, NY, 2011) on all residues having at least one atom within 8 Å of an atom in any of the 20 ligand poses. The Prime refinement starts with a conformational search and minimization of the side chains of the selected residues and after convergence to a low-energy solution, an additional minimization of all selected residues (side chain and backbone) is performed with the truncated-Newton algorithm using the OPLS parameter set and a surface Generalized Born implicit solvent model. The obtained complexes are ranked according to Prime calculated energy (molecular mechanics and solvation), and those within 30 kcal/mol of the minimum energy structure are used in the last step of the process, redocking with Glide 5.7 (Glide, version 5.7, Schrödinger, LLC, New York, NY, 2011) using standard precision, and scoring. In the final round, the ligands used in the first docking step are redocked into each of the receptor structures retained from the refinement step. The final ranking of the complexes is done by a composite score which accounts for the receptor−ligand interaction energy (GlideScore) and receptor strain and solvation energies (Prime energy).

### Flexible Docking Calculations

Flexible Docking Calculations were performed using Macromodel 9.9 (MacroModel, version 9.9, Schrödinger, LLC, New York, NY, 2011). As starting structure we used the best pose derived from IFD calculations for both tautomers of Xanthine (Xan7 and Xan9). Partial charges were calculated using the Jaguar Software (Jaguar, version 7.8, Schrödinger, LLC, New York, NY, 2011). Docking calculations were performed using 1000 steps or 5000 steps search of the mixed Monte Carlo/Low Mode (MC/LMOD) [47] search algorithm with a ratio of 0.5 and OPLSA2005 [48] force field**.** During the LMOD structural perturbation, and during the subsequent energy minimization, all residues within 6.0 Å from the ligand were allowed to move freely. The remaining residues were treated as “frozen atoms.” Additional structural perturbation was applied for all torsion angles of the three “distorted” aminoacids, using the TORS command. The ligand was subjected to explicit translation/rotation with respect to the binding site via the MOLS command available in Macromodel 9.0. Also a distance-dependent dielectric “constant” of 4r was used. After each successful run the complex was minimized using the TNCG algorithm (rmsG <0.01 kJ/mol A). Unique conformations were stored only if they were within the lowest 50 kJ/mol.

### Prgen

Scoring calculations were performed using the PrGen2.1 software according to the following procedure. Theoretical binding affinities are estimated by evaluating ligand-receptor interaction energies, ligand desolvation energies and changes in both ligand-internal energy and ligand internal entropy upon receptor binding: E_binding_ ≈ E_ligand-receptor_ − TΔS_binding_ − ΔG_solvation,ligand_ + ΔE_internal,ligand_. Calculated free energies ΔG°_pred_ are then obtained by linear regression between experimental free energy ΔG°_exp_ and E_binding_. All molecules were superimposed over the position of Xanthine as derived from IFD calculations. Solvation energies, entropy corrections and ligand reference energies were calculated for all ligands after individual minimization using specific built-in PrGen 2.1 modules. To determine the ligand–receptor interaction energy, E_ligand-receptor_, the program uses the force field Yeti _ENREF_48 [49]. Binding affinities are obtained by linear regression between ΔG° and E_binding_. All calculations with PrGen 2.1 were run on a Silicon Graphics Octane.

### Media, Strains and Growth Conditions and Construction of UapA Mutants

Standard complete (CM) and minimal media (MM) for *A. nidulans* were used (http://www.fgsc.net). Auxotrophies were supplemented at the concentrations given in (http://www.gla.ac.uk/acad/ibls/molgen/aspergillus/supplement.html). Nitrogen sources were used at the final concentrations: urea 5 mM, uric acid, adenine or hypoxanthine 0.5 mM. Chemical reagents were obtained from Sigma St. Louis, MO and from AppliChem GmbH. A *ΔuapA ΔuapC ΔazgA argB2 pabaA1* strain transformed with plasmid pAN510-GFP, integrated as a single copy in the *argB* locus, served as a standard wild type control [for details of this strains see 9]. pAN510-GFP carries a fully functional *uapA* gene fused with the *gfp* orf to allow for the subcellular localization of UapA-GFP by epifluorescence microscopy [9], [20]. An isogenic *ΔuapA ΔuapC ΔazgA argB2 pabaA1* mutant was the recipient strain in transformations with mutant *uapA* alleles which were constructed on vector pAN510-GFP by site-directed mutagenesis according to the instructions accompanying the Quik-Change® Site-Directed Mutagenesis Kit (Stratagene), using complementary oligonucleotides carrying the desired substitution (Table S1), Mutations were confirmed by sequencing. The pAN510-GFP vector allows selection of transformants based on arginine auxotrophy complementation [9]. Transformation of *A. nidulans* was as according to Koukaki *et al*. [50]. Transformants expressing intact *uapA*-*gfp* alleles, through single-copy plasmid integration events, were identified by standard PCR and Southern analysis. Growth tests were performed at 25°C and at 37°C, pH 6.8.

### Standard Nucleic Acid Manipulations

Genomic DNA extraction from *A. nidulans* was as described. Plasmid preparation from *E. coli* strains was done with the Nucleospin Plasmid kit according to the manufacturer’s instructions (Macherey-Nagel GmbH). DNA bands were purified from agarose gels using the Nucleospin ExtractII kit according to the manufacturer’s instructions (Macherey-Nagel GmbH). [^32^P]dCTP-labeled molecules used as *uapA* or *argB* specific probes were prepared using a random hexanucleotide primer kit following the supplier’s instructions (Takara Bio Inc.) and purified on MicroSpin™ S-200 HR columns, following the supplier’s instructions (Roche Applied Science). Labeled [^32^P]dCTP (3000 Ci/mmol) was purchased from the Institute of Isotopes Co., Ltd. Restriction enzymes were from Takara Bio Inc. Conventional PCR reactions were done with KAPATaq DNA polymerase (KAPABIOSYSTEMS, USA). Cloning and amplification of products were done with Pfx Platinum (Invitrogen) or Phusion® Flash High-Fidelity PCR MasterMix (New England Biolabs).

### Epifluorescence Microscopy and Transport Kinetic Assays

Samples for fluorescence microscopy were prepared as previously described [9], [14], [20]. In brief, the samples were incubated on coverslips in liquid Minimal Medium supplemented with urea as nitrogen source for 12–14 h at 25°C, observed on an Axioplan Zeiss phase-contrast epifluorescent microscope with appropriate filters, and the resulting images were acquired with a Zeiss MRC5 digital camera using AxioVs40 V4.40.0 software. Images were then processed with Adobe Photoshop CS2 V9.0.2 software. Radiolabelled ^3^H-xanthine (19.6–33.4 Ci/mmol, Moravek Biochemicals, Brea, CA) or 1-^14^C-Lascorbate (2 mCi/mmol, NEN Life Sciences Boston, MA) uptake in conidiospores was assayed at 37°C as described before [12], [20]. *K*_i_ values were calculated from the Cheng and Prusoff equation: *K*_i_  =  IC_50_/(1+L/*K*_m_) where L is the permeant concentration.

## Supporting Information

Figure S1 **Root mean square deviation (RMSD) calculation of the Ca-carbons of all helices, recovering information every 0.25 ns from MD performed for 50 ns.**
(TIF)

Figure S2 **Docking of xanthine analogues in UapA.** (A) 3-methylxanthine, (B) 8-methylxanthine, (C) 9-methylxanthine, (D) 1-methylxanthine, (E) 2-thioxanthine, (F) 6-thioxanthine, (G) 8-azaxanthine, (H) hypoxanthine, (I) adenine, (J) guanine. Hydrogen bonds are depicted with dashed lines. Weak hydrogen bonds are depicted with thin dashed lines.
(TIF)

Table S1 **Forward oligonucleotides used in this study for construction targeted mutation in** ***uapA*** **.** Reverse primers, complementary to the ones listed bellow, were also used.
(DOC)
