# Supplementary figures and images for: Identification of the Substrate Recognition and Transport Pathway in a Eukaryotic Member of the Nucleobase-Ascorbate Transporter (NAT) Family
Source: PLoS One. 2012 Jul 25;7(7):e41939. doi: 10.1371/journal.pone.0041939 (PMC3405029; doi:10.1371/journal.pone.0041939)

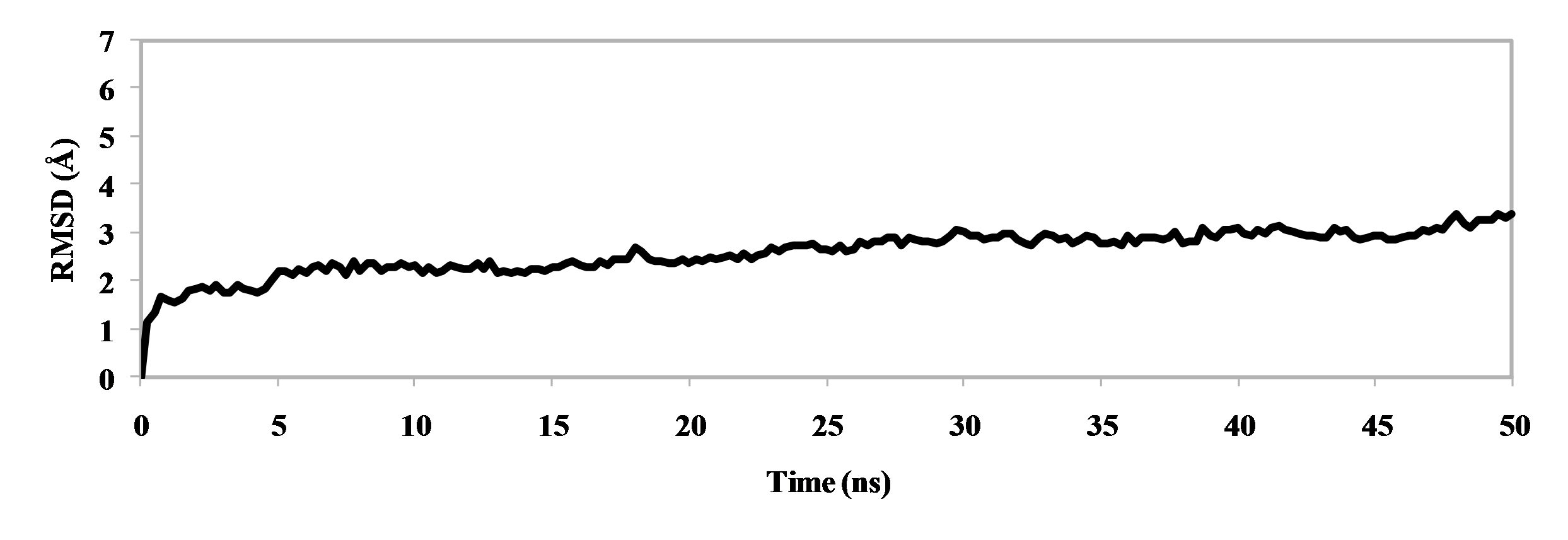

Supplement: Figure S1 — Root mean square deviation (RMSD) calculation of the Ca-carbons of all helices, recovering information every 0.25 ns from MD performed for 50 ns. (TIF) [file pone.0041939.s001.tif]

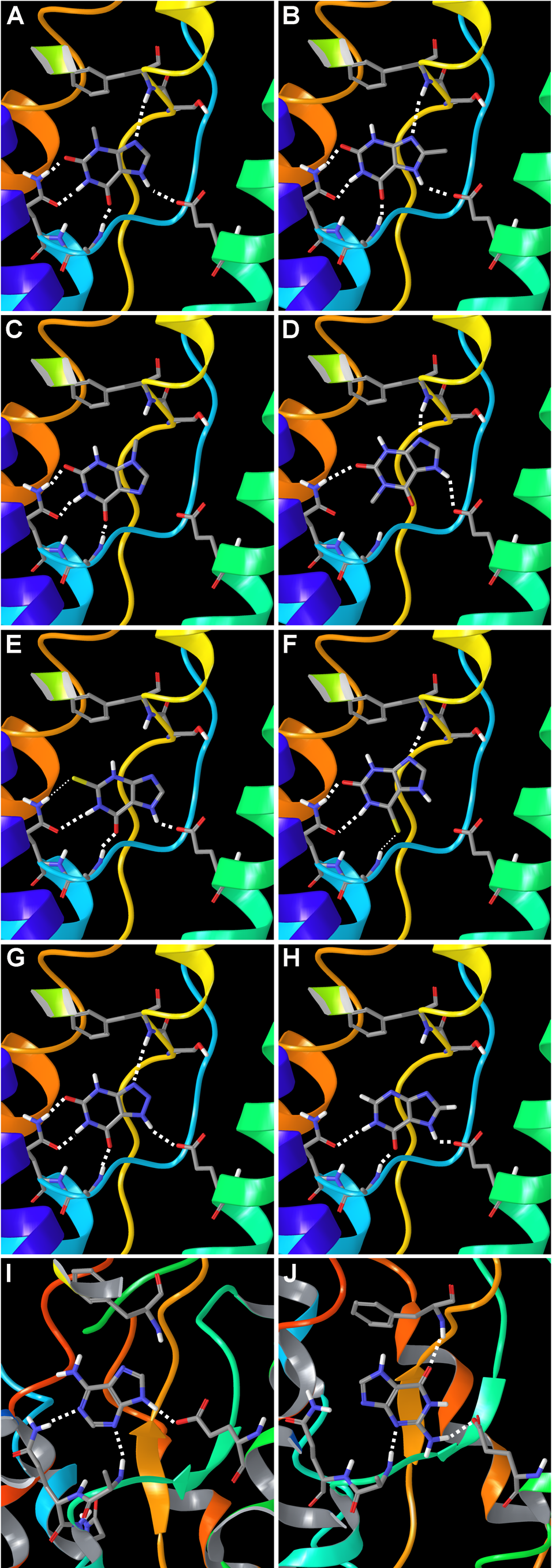

Supplement: Figure S2 — Docking of xanthine analogues in UapA. (A) 3-methylxanthine, (B) 8-methylxanthine, (C) 9-methylxanthine, (D) 1-methylxanthine, (E) 2-thioxanthine, (F) 6-thioxanthine, (G) 8-azaxanthine, (H) hypoxanthine, (I) adenine, (J) guanine. Hydrogen bonds are depicted with dashed lines. Weak hydrogen bonds are depicted with thin dashed lines. (TIF) [file pone.0041939.s002.tif]
