# Supplementary material for: Identification of the Substrate Recognition and Transport Pathway in a Eukaryotic Member of the Nucleobase-Ascorbate Transporter (NAT) Family
Source: PLoS One. 2012 Jul 25;7(7):e41939. doi: 10.1371/journal.pone.0041939 (PMC3405029; doi:10.1371/journal.pone.0041939)
Supplement: Table S1 — Forward oligonucleotides used in this study for construction targeted mutation in uapA . Reverse primers, complementary to the ones listed bellow, were also used. (DOC) [file pone.0041939.s003.doc]

**Table S1.** Primers used for site – directed mutagenesis.

| Mutation | Primer Sequence |
| --- | --- |
| S233A | 5’ – CTGTAATGCTTATCGGGATAGCTCTGATTGGAACTGGGTTC – 3’ |
| T237A | 5’ – CGGGATAAGTCTGATTGGAGCTGGGTTCAAAGACTGGGC – 3’ |
| D360A | 5’ – GCCTGCGAGTGCATCGGTGCTGTAACCGCCACCTGCGACG – 3’ |
| D360H | 5’ – GCCTGCGAGTGCATCGGTCATGTAACCGCCACCTGCGACG – 3’ |
| A407S/Q408P | 5’ – CCCCCATGACGACCTTTTCGCCGAACAACGGCGTG – 3’ |
| T526M/F528A | 5’ – CGAGCTCGTGCTTGAGATGGGGGCTGCGGTCACGGC – 3’ |
| G527V | 5’ – CGAGCTCGTGCTTGAGACGGTCTTTGCGGTCACGGCATTGTAAG – 3’ |
